# Developmental Trajectories of Letter and Speech Sound Integration During Reading Acquisition

**DOI:** 10.3389/fpsyg.2021.750491

**Published:** 2021-11-16

**Authors:** Iliana I. Karipidis, Georgette Pleisch, Sarah V. Di Pietro, Gorka Fraga-González, Silvia Brem

**Affiliations:** ^1^Department of Child and Adolescent Psychiatry and Psychotherapy, University Hospital of Psychiatry Zurich, University of Zurich, Zurich, Switzerland; ^2^Center for Interdisciplinary Brain Sciences Research, Stanford University School of Medicine, Stanford, CA, United States; ^3^Neuroscience Center Zurich, University of Zurich and ETH Zurich, Zurich, Switzerland; ^4^MR-Center of the University Hospital of Psychiatry Zurich, University of Zurich, Zurich, Switzerland

**Keywords:** audiovisual integration, congruency effect, dyslexia, fMRI, children, superior temporal gyrus, ventral occipitotemporal cortex, inferior frontal gyrus

## Abstract

Reading acquisition in alphabetic languages starts with learning the associations between speech sounds and letters. This learning process is related to crucial developmental changes of brain regions that serve visual, auditory, multisensory integration, and higher cognitive processes. Here, we studied the development of audiovisual processing and integration of letter-speech sound pairs with an audiovisual target detection functional MRI paradigm. Using a longitudinal approach, we tested children with varying reading outcomes before the start of reading acquisition (T1, 6.5 yo), in first grade (T2, 7.5 yo), and in second grade (T3, 8.5 yo). Early audiovisual integration effects were characterized by higher activation for incongruent than congruent letter-speech sound pairs in the inferior frontal gyrus and ventral occipitotemporal cortex. Audiovisual processing in the left superior temporal gyrus significantly increased from the prereading (T1) to early reading stages (T2, T3). Region of interest analyses revealed that activation in left superior temporal gyrus (STG), inferior frontal gyrus and ventral occipitotemporal cortex increased in children with typical reading fluency skills, while poor readers did not show the same development in these regions. The incongruency effect bilaterally in parts of the STG and insular cortex at T1 was significantly associated with reading fluency skills at T3. These findings provide new insights into the development of the brain circuitry involved in audiovisual processing of letters, the building blocks of words, and reveal early markers of audiovisual integration that may be predictive of reading outcomes.

## Introduction

Reading is acquired over the course of many years and extensive practice is required to achieve fluent and efficient text reading competence and comprehension skills. Alphabetic writing systems are based on the principle that each speech sound corresponds to one or a combination of printed characters, namely letters. This process of mapping speech sounds to letters is taught at the very beginning of formal reading instruction and is a prerequisite for decoding sublexical units, such as syllables, bigrams, and trigrams, and eventually for the recognition of word forms. However, insights into how children’s brains develop during the acquisition of culturally defined character-speech sound associations and how specific areas in the auditory and visual processing system adapt to process letter-speech sound combinations as audiovisual concepts are still sparse.

Parts of the auditory cortex and superior temporal regions have been identified as the main audiovisual integration site for words (McNorgan et al., 2014), as well as for letters and speech sounds (Raij et al., 2000; van Atteveldt et al., 2004). Letter-speech sound integration is a fast, automated process with electrophysiological responses characteristic to audiovisual processing arising as early as 150 ms (mismatch negativity; Froyen et al., 2009) but also extending to later multisensory integration processes at 380–540 ms (superior temporal sulcus (STS) activation, Raij et al., 2000) and around 650 ms after stimulus presentation (late negativity, Žarić et al., 2014). During letter-speech sound processing, expert readers of transparent and semi-transparent alphabetic systems have been found to engage superior temporal brain areas more strongly when speech sounds are paired with congruent letters compared to incongruent letters (Raij et al., 2000; van Atteveldt et al., 2004; Blau et al., 2009). A similar congruency effect was also observed in the Heschl’s gyrus of 9-year-old typical readers (Blau et al., 2010), while adolescent readers with typical reading skills showed the opposite pattern, characterized by stronger responses for incongruent than congruent print-speech pairs in the left superior temporal gyrus (STG; Kronschnabel et al., 2014).

Letter-speech sound integration has been shown to rapidly develop at a very early stage of reading acquisition and is related to reading outcomes (Frost et al., 2009; Preston et al., 2016; Chyl et al., 2018). Already prereaders showed effects of audiovisual integration after a short artificial letter-speech sound training, which depended on their learning rate (Karipidis et al., 2017). Fast learners showed stronger congruency effects for trained artificial letter speech sound pairs in the right STG and left inferior temporal cortex. In addition, audiovisual integration in the left planum temporale (PT) of prereading children was significantly related to future reading fluency outcomes (Karipidis et al., 2018). Learning audiovisual correspondences also induced changes in the visual processing of artificial letters in text-selective regions of left ventral occipitotemporal cortex (vOTC), located in the posterior fusiform and occipitotemporal sulcus (OTS), which were dependent on the training performance of the preschoolers (Pleisch et al., 2019a).

Specific portions of vOTC located along the middle and posterior OTS are commonly referred to as the visual word form area(s) (VWFA) and selectively respond to words, letters, and other print stimuli (Cohen et al., 2002; McCandliss et al., 2003; Lerma-Usabiaga et al., 2018; Caffarra et al., 2021a). This visual specialization emerges rapidly when children learn how to read and is refined over the course of reading acquisition. It has been shown that children (Brem et al., 2010; Pleisch et al., 2019a) and adults (Madec et al., 2016) show increased activation in text-selective portions of vOTC after intensive grapho-phonological training. In beginning readers, auditory processing with high phonological awareness demands also engages parts of vOTC, activation of which depends on reading ability (Wang et al., 2018). Audiovisual processing of letters and speech sounds engages left vOTC more than other audiovisual stimuli, such as numerals and number names (Holloway et al., 2015). Activation in vOTC during audiovisual processing of letter-speech sound pairs also depends on reading ability and has been found to be reduced in dyslexia (Richlan, 2019; Romanovska et al., 2021). Effects of audiovisual congruency have been reported less consistently for vOTC. In a sample of adolescent readers, Kronschnabel et al. (2014) reported an incongruency effect for letter-speech sound pairs and short pseudowords in left vOTC for typical readers, while poor readers showed effects toward a congruency effect.

Despite the increasing interest in studying print and speech processing in early stages of development, longitudinal studies covering multiple time points during the course of reading acquisition are still very scarce (Chyl et al., 2021). We recently reported first longitudinal evidence showing a positive association between congruency effects for non-word stimuli in the left STG and improvement in reading skills from first to second grade (Wang et al., 2020). In addition, a recent magnetoencephalography (MEG) study showed in a cross-sectional and longitudinal cohort that an electrophysiological incongruency effect for syllables emerges from prereading to early reading stages, stemming from the left superior temporal cortex (Caffarra et al., 2021b). An earlier MEG study found that beginning readers show an audiovisual processing effect for letters and speech sounds in temporoparietal sources and this effect correlated with literacy skills (Xu et al., 2018).

However, it remains unclear how audiovisual processing of letter-speech sound pairs changes from the prereading to the early reading stages and how it is associated with reading development. Automated retrieval of correspondences between letters and speech sounds is a prerequisite for successful reading acquisition (Ziegler and Goswami, 2005). One of the leading theories of dyslexia postulates that difficulties in crossmodal integration can lead to an impairment in the automatization of grapho-phonological entities (Blomert, 2011). Deficits in print-speech automaticity could also be driven by difficulties in selectively processing linguistic information or poor phonological and language skills, which often characterize young struggling readers (Pennington et al., 2012). Considering audiovisual integration of letters and speech sounds as a sensory process that develops during reading acquisition, presumably by engaging brain regions that are specialized for auditory, visual, and cross-modal processing, understanding its development could help explain neurobiological mechanisms that influence reading acquisition.

The aim of the current study was to investigate developmental trajectories of neural activation to letter-speech sound pairs in a group of children with varying risk for developmental dyslexia and reading outcomes. We focused on analyzing longitudinal fMRI data during an audiovisual target detection task at three crucial stages: (1) before the start of formal reading instruction (at the end of second year of kindergarten), (2) at the middle of first grade, when full letter knowledge is almost attained but reading is still imprecise and sluggish, and (3) at the middle of second grade, when accurate reading is expected but reading fluency is still being practiced intensively. Additionally, we investigate how development of audiovisual letter-speech sound processing relates to children’s reading outcomes.

## Materials and Methods

### Participants

A sample of 50 German-speaking children completed the fMRI experiment presented here at least on one of the following three time points: at T1, within 4 months prior to the start of formal reading acquisition (kindergarten), at T2, 5–9 months after the start of formal reading acquisition (grade 1), and at T3, 5–9 months after the start of the second year of formal reading acquisition (grade 2). The data of three participants was excluded due to poor data quality at all available time points. From the remaining 47 participants, *n* = 29 met the stringent data quality criteria for all three time points and eighteen had no available data in at least one of the time points due to the following reasons: one only participated at T1, six discontinued participation or wore braces at T3, for two participants data were excluded due to poor data quality at T1, and additional nine had no available data for T1 because they were enrolled to the study at T2. The subsample of *n* = 29 with complete longitudinal fMRI data served as the core sample for the whole-brain analyses, while the enlarged sample of *n* = 47 (*n*_T1_ = 36; *n*_T2_ = 45; *n*_T3_ = 40) was used for region of interest (ROI) analyses that permitted missing values (Table 1).

**TABLE 1:** Participant characteristics.

	Core sample (*n* = 29)			Enlarged sample (*n* = 47)		
	Typical	Poor	Group statistics	Typical	Poor	Group statistics
Sex (f/m)	8/11	6/4	χ^2^ = 0.84 *p* = 0.359	14/16	10/7	χ^2^ = 0.64 *p* = 0.423
Handedness(R/L)	16/3	10/0	χ^2^ = 1.76 *p* = 0.184	26/4	16/1	χ^2^ = 0.63 *p* = 0.426
ARHQ^a^	0.49 ± 0.17	0.61 ± 0.10	*t* = −2.53 *p* = 0.017	0.47 ± 0.15	0.56 ± 0.12	*t* = −1.99 *p* = 0.053
IQ	102.68 ± 10.48	101.60 ± 9.42	*t* = 0.27 *p* = 0.786	102.10 ± 10	100.82 ± 8	*t* = 0.45 *p* = 0.655
Age T1	6.68 ± 0.32	6.63 ± 0.32	*t* = 0.44 *p* = 0.660	6.66 ± 0.31	6.62 ± 0.29	*t* = 0.32 *p* = 0.748
Age T2	7.32 ± 0.31	7.31 ± 0.30	*t* = 0.09 *p* = 0.925	7.32 ± 0.30	7.38 ± 0.36	*t* = −0.57 *p* = 0.570
Age T3	8.44 ± 0.32	8.37 ± 0.30	*t* = 0.58 *p* = 0.567	8.40 ± 0.31	8.43 ± 0.33	*t* = −0.28 *p* = 0.783
Letter-speech sound knowledge T1^b^	16.00 ± 11.64	11.60 ± 8.97	*t* = 1.04 *p* = 0.307	16.96 ± 11.88	12.42 ± 8.36	*t* = 1.19 *p* = 0.241
Word and pseudoword reading fluency T3^c^	48.68 ± 19.51	7.60 ± 5.70	*t* = 7.54 *p* < 0.001	48.64 ± 17.56	7.16 ± 5.03	*t* = 10.17 *p* < 0.001

*Values are mean ± SD ^a^Highest parental ARHQ score: 22 children of the core and 34 children of the enlarged sample exceeded the ARHQ risk score of > 0.4, indicating a considerable familial risk for developmental dyslexia; ^b^raw values: n; ^c^percentile scores.*

This sample was drawn from a large longitudinal study focusing on cognitive and brain development of children at varying familial risk for developmental dyslexia over multiple time points during the course of reading acquisition (Karipidis et al., 2017, 2018; Pleisch et al., 2019a, b; Mehringer et al., 2020; Wang et al., 2020; Fraga-González et al., 2021). Familial risk for dyslexia was estimated using the Adult Reading History Questionnaire (ARHQ; Lefly and Pennington, 2000). Two participants of the enlarged sample were treated for attention deficit/hyperactivity disorder and discontinued their medication for 48 h before all neuroimaging sessions and behavioral testing. All participants reported no other neurological or psychiatric disorders, had normal visual and auditory acuity, and had a non-verbal IQ-estimate of above 80. The study was approved by the ethics committee of the Kanton of Zurich and neighboring cantons in Switzerland. All assessments and experiments were undertaken with the understanding and written consent of a legal guardian and oral consent of all children.

### Neurocognitive and Reading Assessments

An extensive neurocognitive test battery was performed at all-time points (Table 1). Letter sound knowledge was tested for all upper- and lower-case letters of the Latin alphabet, as well as for the umlaut vowels of German (ä, ö, ü). Letter-sound knowledge scores only showed meaningful variability at T1, with children reaching ceiling performance at T2 and T3. Word and pseudoword reading fluency were tested using the Salzburger Lese- und Rechtschreibtest at T2 and T3 (SLRT-II, Moll and Landerl, 2010). For T3, age-adjusted standardized scores for word and pseudoword reading were averaged to compute the reading fluency outcome score. Participants with a mean reading fluency score below the 16th percentile were classified as poor readers (*n* = 10 for the core sample; *n* = 17 for the enlarged sample). Non-verbal IQ was assessed using the CFT1-R (Weiß and Osterland, 2013).

### Experimental Paradigm

The participants performed an implicit audiovisual target detection task at all-time points (Kronschnabel et al., 2014; Karipidis et al., 2017). The task was programmed using Presentation^®^ (Version 16.4)^1^ and included four conditions: congruent and incongruent pairs of single letter-speech sound correspondences, as well as unimodally presented letters and speech sounds. The current analysis focuses on the fMRI data of the audiovisual conditions (for analyses of the visual condition see Pleisch et al., 2019a; Fraga-González et al., 2021).

**FIGURE 1:**
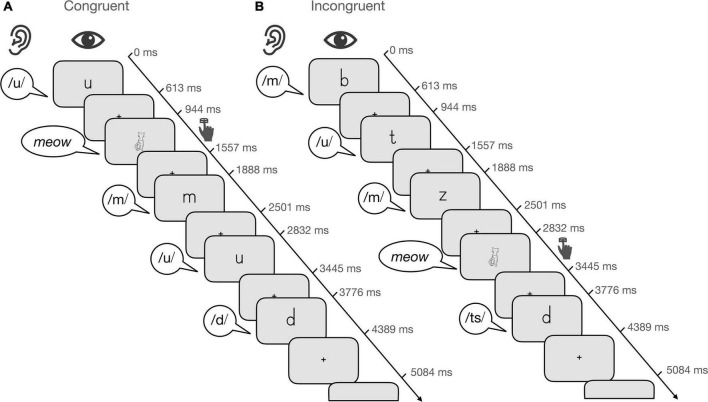
Audiovisual target detection task. Illustration of five trials for **(A)** the audiovisual congruent condition and **(B)** the audiovisual incongruent condition. Each block consisted of 15 trials that were presented pseudorandomly for 613 ms with an interstimulus interval of 331 or 695 ms. Participants were instructed to respond when the target, i.e., the drawing of a cat appeared.

The task consisted of 16 blocks (4 blocks/condition) and total task duration was 375 s. Unimodal and bimodal blocks (15 trials/block) alternated pseudorandomly and were separated by fixation periods of 6 or 12 s. Each condition included 54 experimental trials and 6 target trials. The trials within each block were presented pseudorandomly for 613 ms with an interstimulus interval of 331 or 695 ms (Figure 1). Visual information was presented over video goggles (VisuaStimDigital, Resonance Technology, Northride, CA) and auditory information over in-ear headphones (MR confon GmbH, Magdeburg). Letters were presented in black in the middle of a gray background (mean visual angle: horizontally 2.8°; vertically 4.8°). Participants were instructed to respond by button press to the target, which was the drawing or sound of a cat, or the audiovisual presentation of both.

Accuracy and reaction times were analyzed using linear mixed models. Accuracy in target detection was high, 93.4 ± 6.2% for the core sample and 94.0 ± 6.5% for the enlarged sample, with a mean reaction time of 677 and 674 ms, respectively. Accuracy did not significantly differ between the three time points [ACC_core_: *F*_(2, 83)_ = 1.71, *p* = 0.188; ACC_enlarged_: *F*_(2, 117)_ = 0.71, *p* = 0.494]. As expected, reaction times decreased over time, i.e., children responded significantly faster as they grew older [RT_core_: *F*_(2, 83)_ = 13.68, *p* < 0.001; RT_enlarged_: *F*_(2, 117)_ = 11.57, *p* < 0.001]. Responses of one participant at T1 were not logged due to a technical problem and therefore not included in the response analysis.

### MRI Data Acquisition and Preprocessing

MRI data was recorded on a Philips Achieva 3 Tesla scanner (Best, The Netherlands) using a 32-element receive head coil. Using a T2*-weighted whole-brain gradient-echo planar image sequence, 189 volumes were acquired during a simultaneous EEG-fMRI recording. The following acquisition parameters were used: slices/volume: 31, repetition time: 1.98 s, echo time: 30 ms, slice thickness: 3.5 mm, slice gap: 0.5 mm, flip angle: 80°, field of view: 240 × 240 mm^2^, in-plane resolution: 3 × 3 mm^2^, SofTone factor: 3, sensitivity-encoding (SENSE) reduction factor: 2.2. In addition, a field map and a high-resolution T1-weighted anatomical image were acquired.

FMRI data was preprocessed and analyzed using SPM12. Preprocessing included B0 field map correction, realignment and unwarping, slice time correction, coregistration and segmentation, normalization, resampling (3 × 3 × 3 mm^3^), smoothing (8 mm FWHM), and normalization to Montreal Neurological Institute (MNI) standard space based on deformations derived from the segmentation and a pediatric anatomical template (age range 5.9–8.5 years) created using the Template-OMatic toolbox (Wilke et al., 2008).

After preprocessing, movement artifact correction was performed as implemented in the ArtRepair toolbox (Mazaika et al., 2007). Motion affected volumes with scan-to-scan movement of more than 1.5 mm were repaired using linear interpolation between the nearest unrepaired scans. If more than 15% of the scans needed to be repaired, the data was excluded from further analysis. In addition, if a scan was preceded and followed by a motion affected scan or if more than two consecutive scans were affected by movement, scrubbing was performed by modeling the affected volumes in a binary regressor of no interest (for details see Supplementary Material).

### Whole-Brain fMRI Analysis

The whole-brain analysis focused on the development of audiovisual processing of single letters and speech sounds and was performed using the core sample (*n* = 29). We calculated a whole-brain ANOVA with factors time (T1, T2, and T3) and congruency (congruent and incongruent) to test for developmental effects of audiovisual integration. In addition, familial risk for dyslexia, letter-sound knowledge at T1 and individual reading fluency scores at T3 were used to perform multiple regression analyses with whole-brain activation of each condition within each time point. All whole-brain analyses were restricted to a gray matter mask which included all voxels that were classified as gray matter volume with a probability of > 0.5 in the tissue probability map of the pediatric MNI template. We applied a voxel-wise uncorrected threshold of *P* < 0.001 with a cluster size threshold of k > 15. We also report cluster-level corrected *P*-values (*P* < 0.05). Results that are not significant after correction for multiple comparisons should be interpreted with caution and need to be replicated.

### Region of Interest Analysis

**FIGURE 2:**
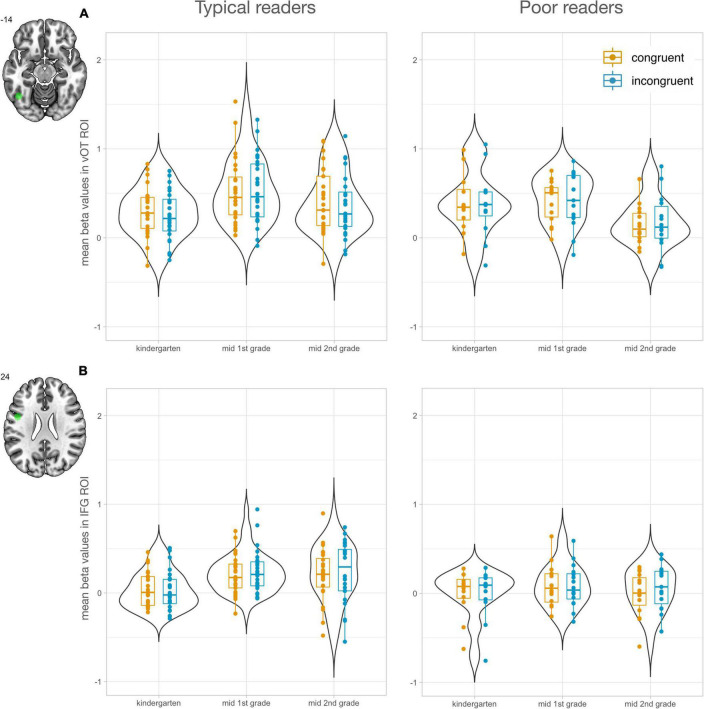
Letter-specific region of interest analyses (*n* = 47). Left panels show values for typical readers and right panels for poor readers. Mean responses to congruent letter-speech sound pairs are shown in orange and to incongruent in blue. **(A)** Mean beta values in left vOTC ROI increased from kindergarten (T1) to 1st grade (T2) and second grade (T3) in the typical reading group, while the poor reading group showed a significant decrease from 1st grade (T2) to second grade (T3). **(B)** Mean beta values in left IFG ROI increased from kindergarten (T1) to 1st grade (T2) and second grade (T3) in the typical reading group. IFG activation was significantly higher for typical readers than poor readers at T3.

**FIGURE 3:**
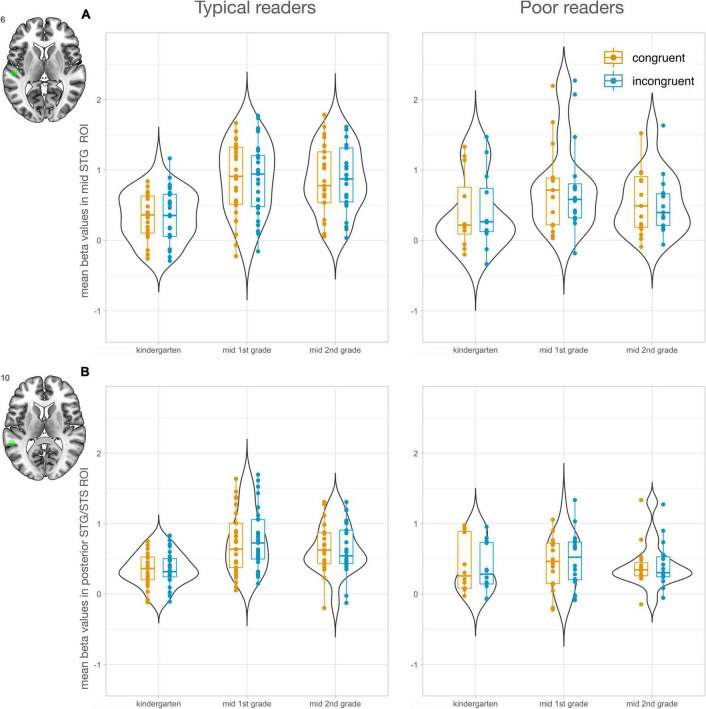
Region of interest analyses in audiovisual processing areas of the STG (*n* = 47). Mean beta values in STG ROIs increased from kindergarten (T1) to 1st grade (T2) and second grade (T3) in the typical reading group. **(A)** Mid STG ROI (midSTG). **(B)** Posterior STG ROI (postSTG). Left panels show values for typical readers and right panels for poor readers. Mean responses to congruent letter-speech sound pairs are shown in orange and to incongruent in blue.

To investigate the development of letter processing in key regions of reading and audiovisual processing, region of interest (ROI) analyses were performed. ROIs were selected using the meta-analysis tool of NeuroSynth (Yarkoni et al., 2011). The search term “letter” yielded two peaks, one in the vOTC (*x* = −44, *y* = −60, *z* = −14) and one in the IFG (*x* = −46, *y* = 2, *z* = 24; Figure 2). In addition, the search term “audiovisual” revealed two peaks in the STG, a mid STG ROI (midSTG: *x* = −52, *y* = −22, *z* = 6) and a posterior ROI in the STG/STS (postSTG: −56, −42, 10; Figure 3). The midSTG ROI falls within the primary auditory cortex, while the postSTG ROI includes parts of the STS and represents audiovisual integration regions (Blau et al., 2009; Holloway et al., 2015). Each ROI was defined as a 6mm radius sphere around the peak coordinates, which are provided in MNI space.

For each ROI, we calculated a linear mixed model (LMM) with factors time (T1, T2, T3), reading fluency at T3 (typical, poor), and congruency (congruent, incongruent). The enlarged sample (*n* = 47) was used for these analyses, given that LMM can handle missing data points. Standardized residuals were used to identify and exclude outliers deviating more than 3 standard deviations from the mean. For significant interaction effects, *post hoc t*-tests were computed, and Tukey Kramer corrected *P*-values are reported. We also tested for associations of audiovisual integration at each time point with familial risk for dyslexia, letter-sound knowledge at T1, and reading fluency outcome at T3. Individual differences in processing incongruent and congruent letter-speech sound pairs in each ROI were used as a measure for audiovisual integration and were correlated with each of the behavioral measures (*P* < 0.05).

LMM with factors time and reading were also computed using the incongruency effect (Supplementary Figures 4, 5). In addition, supplementary ROI analyses were performed to replicate the vOTC and STG effects in functionally defined ROIs (Supplementary Figures 2, 3).

## Results

### Whole-Brain Analyses

**FIGURE 4:**
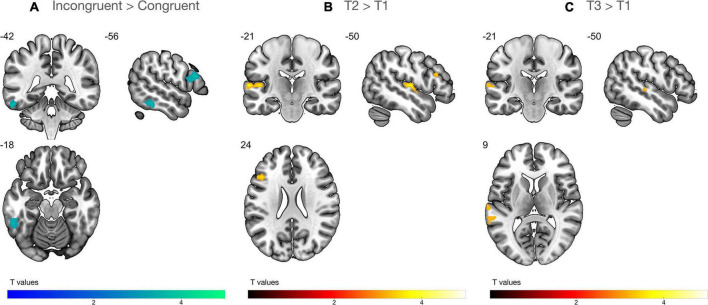
Whole-brain analysis (*n* = 29). **(A)** Incongruency effect: higher activation for incongruent than congruent letter-speech sound pairs in the left inferior frontal gyrus and the left ventral occipitotemporal cortex. **(B)** Effect of time: stronger audiovisual processing at T2 than T1 in the left inferior frontal gyrus and superior temporal gyrus/planum temporale. **(C)** Effect of time: activation increase for audiovisual processing in the left superior temporal gyrus is still evident at T3.

The ANOVA (*n* = 29) with factors time (T1, T2, T3) and congruency (congruent, incongruent) showed that audiovisual processing of single letter-speech sound pairs elicited strong blood oxygen level dependent (BOLD) responses in large portions of vOTC and STG, as well as in the inferior frontal gyrus (IFG), middle frontal gyrus (MFG), superior parietal lobule (SPL), and angular gyrus (AnG; Supplementary Figure 1 and Supplementary Table 1). We found a significant main effect of congruency that was characterized by stronger BOLD responses for incongruent than congruent pairs in the left IFG and left vOTC across all time points (Figure 4A). In addition, brain activation in the left IFG and STG, including parts of the planum temporale (PT) significantly increased from T1 to T2 during audiovisual processing of letter-speech sound pairs (Figure 4B). Audiovisual processing of letter-speech sound pairs was also stronger in the left STG at T3 compared to T1 (Figure 4C and Table 2).

**TABLE 2:** Whole-brain analysis (*n* = 29).

Brain area	MNI coordinates			Voxels	*T*-value	Peak-level P uncor	Cluster-level P uncor	Cluster-level P FWEcorr
	x	y	z					
**Incongruent > congruent**								
IFG left	−56	21	21	41	3.86	<0.0001	0.035	0.273
ITG/vOTC left	−59	−42	−24	36	3.70	0.0001	0.046	0.344
**T2 > T1**								
STG/PT left	−59	−30	6	85	4.23	<0.0001	0.004	0.038*
IFG left	−47	24	21	22	3.70	0.0001	0.108	0.632
**T3 > T1**								
STG left	−68	−18	9	20	3.67	0.0002	0.124	0.682
STG left	−65	−39	9	17	3.44	0.0004	0.154	0.758
**Incongruent > congruent T1 × letter knowledge T1**								
STG/PP left	−47	3	−6	16	4.48	<0.0001	0.160	0.781
**Incongruent > congruent T1 × reading ability T3**								
STG/insula	40	−3	0	32	4.77	<0.0001	0.050	0.390
	−32	0	12	20	4.34	<0.0001	0.112	0.669
**Congruent > incongruent T2 × reading ability T3**								
AnG left	−47	−54	48	16	4.06	0.0002	0.120	0.752

*Results were masked using a gray matter mask. Voxel-wise uncorrected threshold of P < 0.001, k > 15. Asterisk marks cluster-level FWE corrected P < 0.05.*

*IFG, inferior frontal gyrus; ITG, inferior temporal gyrus; vOTC, ventral occipitotemporal cortex; PT, planum temporale; STG, superior temporal gyrus; PP, planum polare; AnG, angular gyrus.*

**FIGURE 5:**
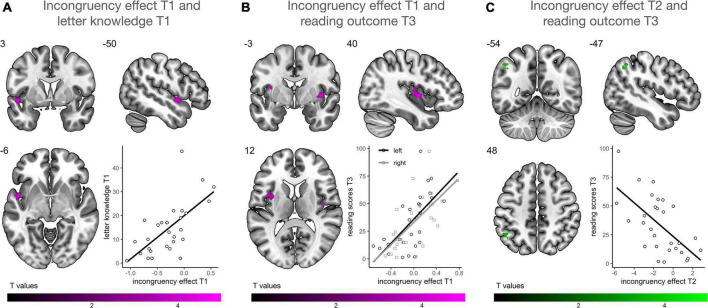
Multiple regression analysis (*n* = 29). **(A)** Higher activation for incongruent than congruent letter-speech sound pairs in the left anterior superior temporal gyrus (STG)/planum polare at T1 was associated with higher letter knowledge at T1. **(B)** Higher activation for incongruent than congruent letter-speech sound pairs bilaterally in the left STG and insula at T1 was associated with higher reading fluency scores at T3. **(C)** Higher activation for incongruent than congruent letter-speech sound pairs in the left angular gyrus at T2 was associated with lower reading fluency scores at T3.

Using multiple regression analysis, we investigated whether audiovisual integration at each time point, reflected by the incongruency effect (incongruent vs. congruent), was associated with familial risk for dyslexia, letter knowledge at T1 and reading outcomes at T3. We found no association between individual risk for dyslexia and the strength of the incongruency effect on a whole brain level. Prereading children with higher letter-sound knowledge at T1 showed a stronger incongruency effect in the left planum polare (PP), the anterior portion of the STG (Figure 5A). Particularly children with low letter knowledge showed higher neural responses for congruent than incongruent letter-speech sound pairs in this region. A stronger incongruency effect at T1 bilaterally in a more posterior portion of the STG, extending to parts of the posterior insular cortex, was significantly associated with higher reading fluency scores at T3 (Figure 5B). Finally, a stronger incongruency effect in the left angular gyrus (AnG) at T2 was associated with lower reading fluency scores at T3 (Figure 5C; Table 2), i.e., children with better reading fluency scores at T3 showed stronger responses to congruent than incongruent letter-speech sound pairs in the left AnG.

### Region of Interest Analysis

#### Letter-Speech Sound Processing in Letter-Specific Regions of Interest

Using the meta-analysis tool Neurosynth with the search term “letter,” we identified two ROIs that previously showed letter-specific activation, one in the left vOTC and one in the left IFG (Figure 2). The LMM with factors time, congruency, and reading fluency was computed using mean beta values in these ROIs.

For the left vOTC ROI, we found a main effect of time [*F*_(2, 176)_ = 13.07, *P* < 0.001; Figure 2A]. Activation in left vOTC significantly increased from T1 to T2 [*t*(176) = 3.90, Pcor < 0.001] and decreased from T2 to T3 [*t*(176) = 4.65, Pcor < 0.001]. The significant interaction of time and reading ability [*F*_(2, 176)_ = 8.27, *P* < 0.001] indicated that this developmental effect showed distinct developmental trajectories based on reading outcome (Figure 2A). Activation in left vOTC during audiovisual processing of letters only increased in children with typical reading outcomes from T1 to T2 [*t*(176) = 5.15, Pcor < 0.001], a developmental increase that was still evident in T3 [*t*(176) = 2.93, Pcor = 0.043]. Children with poor reading outcomes did not show a significant increase of activation in left vOT from T1 to T2 [*t*(176) = 1.10, Pcor = 0.879] but a decrease at T3 [T1 > T3: t(176) = 2.90, Pcor = 0.048; T2 > T3: *t*(176) = 4.24, Pcor = 0.001], which probably drove the reduction of activation observed in the main effect for T3. Even though the two groups showed diverging developmental patterns, group differences within time points were not significant (Pcor > 0.121). A supplementary analysis revealed that the incongruency effect in left vOTC increased over time (Supplementary Figure 4A). We found no significant correlations between incongruency effects and letter-sound knowledge at T1, reading fluency outcome at T3 or familial risk for dyslexia.

The LMM in the left IFG revealed a significant main effect of time [*F*_(2, 183)_ = 6.32, *P* = 0.002; Figure 2B]. Audiovisual processing in the left IFG increased after the start of formal reading instruction and was significantly stronger for T2 > T1 [*t*(183) = 3.42, Pcor = 0.002] and T3 > T1 [*t*(183) = 2.73, Pcor = 0.019]. The interaction between time and reading ability showed that this developmental increase was specifically evident in typical readers [T2 > T1: *t*(183) = 3.91, Pcor = 0.002; T3 > T1: *t*(183) = 3.86, Pcor = 0.002] and not in poor readers (*P* > 0.732). In addition, at T3 the typically reading group showed significantly stronger responses in the left IFG compared to the poorly reading group [*t*(183) = 2.93, Pcor = 0.044]. In line with the whole-brain analysis, supplementary results focusing on the incongruency effect in the IFG showed an increase of incongruent vs. congruent activation over time (Supplementary Figure 4B). Incongruency effect in the left IFG ROI was not significantly correlated with familial risk for dyslexia, letter-sound knowledge at T1, and reading fluency outcome at T3.

#### Letter-Speech Sound Processing in Audiovisual Regions of Interest

The search term “audiovisual” in NeuroSynth resulted in two peaks along the STG/STS. We found a significant main effect of time for both STG ROIs [midSTG: *F*_(2, 185)_ = 16.51, *P* < 0.001; postSTG: *F*_(2, 185)_ = 10.77, *P* < 0.001]. STG activation increased over time particularly from T1 to T2 [midSTG: *t*(185) = 5.71, Pcor < 0.001; postSTG: t(185) = 4.59, Pcor < 0.001], and from T1 to T3 [midSTG: *t*(185) = 3.75, Pcor < 0.001; postSTG: *t*(185) = 3.20 Pcor < 0.005]. No significant main effect of reading was found for midSTG [*F*_(1, 185)_ = 1.67, *P* = 0.197], while postSTG showed group differences on a trend level [*F*_(1, 185)_ = 3.76, *P* = 0.054]. The interaction of time and reading ability was significant for both STG ROIs [midSTG: *F*_(2, 185)_ = 4.66, *P* = 0.011; postSTG *F*_(2, 185)_ = 5.69, *P* = 0.004] and revealed that audiovisual processing in the STG particularly increased in the typical reading group [midSTG: T2 > T1 *t*(185) = 6.29, Pcor < 0.001, T3 > T1 *t*(185) = 5.75, Pcor < 0.001; postSTG: T2 > T1 *t*(185) = 6.64, Pcor < 0.001, T3 > T1 *t*(185) = 5.00, Pcor < 0.001] and not in the poor reading group (*P* > 0.129). The typical and poor reading groups showed the strongest difference at T2 for the postSTG ROI [*t*(185) = 2.81, Pcor = 0.061] and at T3 for the midSTG ROI [*t*(185) = 2.67, Pcor = 0.086].

The supplementary analysis, focusing on the development of the incongruency effect, only revealed a developmental change of incongruent vs. congruent activation in the postSTG ROI (Supplementary Figure 5). The strongest incongruency effect in the postSTG ROI was evident at T2 (Supplementary Figure 5B). We found no significant correlations between incongruency effects in the two STG ROIs and letter-sound knowledge at T1, reading fluency outcome at T3 and familial risk for dyslexia.

## Discussion

Here, we investigated the development of audiovisual letter-speech sound processing and integration from prereading to early reading stages by acquiring longitudinal fMRI data in a group of children before the start of formal reading acquisition (T1), in the middle of first grade (T2) and in the middle of second grade (T3). We found that after the start of reading acquisition at T2, brain activation to audiovisual letter presentations increases in the STG, IFG, and vOTC, a network of regions that is involved in orthographic and phonological processing of written language (Richlan, 2019). This developmental increase was particularly pronounced for children with typical reading abilities in second grade. In addition, effects of audiovisual integration, measured as the incongruency effect between matching and non-matching audiovisual letter presentations, were found in the left vOTC and IFG and appeared to show only marginal changes over time. Interestingly, stronger incongruency effects in bilateral parts of the STG and posterior insula at T1 were associated with higher reading fluency levels at T3. Overall, these results suggest that neural responses to audiovisually presented letters rapidly change in the first 2 years of reading acquisition in line with the behavioral improvements in letter knowledge and the gains in reading skills during this developmental stage. Particularly typical readers showed the strongest developmental increase in audiovisual processing from kindergarten (T1) to first grade (T2) while poor readers showed a different developmental trajectory in the target regions, with hardly any differences, paralleling their reading expertise.

The whole-brain analysis revealed that the strongest developmental effects of letter-speech sound processing from T1 to T2/T3 were located in the left STG. Reading acquisition leads to increased activation in brain regions involved in phonological processing, including the superior temporal cortex (Monzalvo and Dehaene-Lambertz, 2013). Our results suggest that after a few months of reading instruction audiovisual processing in the left STG increases. Examining two ROIs in the STG revealed that this developmental effect was evident in children who eventually had typical reading skills at the middle of second grade (T3). However, children who would develop poor reading skills did not show significant increases in STG activations from T1 to T2/T3. In addition, lower activation was observed in the posterior STG/STS in poor beginning readers, with the strongest group difference evident at T2, when posterior STG/STS activation was higher for typical than poor readers on a trend level. Therefore, the most pronounced group difference of audiovisual processing in the left STG/STS between typical and poor readers was found in the middle of first grade, when letter-speech sound correspondences are intensively trained but are not yet fully automatized.

A previous fMRI study focusing on beginning readers reported that STS activation to speech and print positively correlated with word reading skills (Chyl et al., 2018). Our experimental paradigm allowed us to also investigate how effects of audiovisual integration are related to reading skills. Stronger incongruency effects bilaterally in the STG and parts of the posterior insula at the prereading stage were associated with future reading skills 2 years later (T3). Thus, early markers of audiovisual integration in primary and associative auditory regions may be predictive of individual reading development. In older children, congruency effects in the auditory cortex have been found to increase as a function of literacy skills (Blau et al., 2010; McNorgan et al., 2014). The direction of the congruency effect shows extensive inconsistencies in the literature that are likely caused by differences in temporal and spatial resolution of the applied neuroimaging methods (fMRI vs. EEG/MEG; Caffarra et al., 2021b), attentional demands of the experimental paradigms [e.g., synchronous vs. asynchronous audiovisual presentation (van Atteveldt et al., 2007); implicit vs. explicit], stimulus material (letters, syllables, pseudowords or words; Kronschnabel et al., 2014), different levels of transparency in the studied alphabetic languages (Holloway et al., 2015; Xu et al., 2019), and the varying age-ranges of the samples (Wang et al., 2020).

We were also interested in whether audiovisual integration effects in our sample were related to individual familial risk for dyslexia. Familial history of dyslexia has been reported to influence phonemic representations in temporal regions and audiovisual integration in the left superior temporal cortex at early reading stages (Plewko et al., 2018; Vandermosten et al., 2020). In an fMRI study, Polish-speaking children with low familial risk showed an incongruency effect for letter speech sound pairs, while children with increased familial risk for dyslexia showed a congruency effect (Plewko et al., 2018). We were not able to replicate this finding in children of a slightly less transparent language i.e., German. However, we also show that in typical reading development an early incongruency effect emerges in superior temporal regions. Plewko et al. (2018) argue that the incongruency effect in the left STC is characteristic for beginning readers and it might reverse into a congruency effect later, when letter-speech sound pairs are automatized. Their study showed that children at a very early reading stage who later developed dyslexia showed higher activation in the STC for congruent letter speech sound pairs than future typical readers (Plewko et al., 2018). This is in line with our findings, given that a higher congruency effect in the STG at T1 was associated with lower reading skills at T3. Larger longitudinal studies are needed to clarify if the initial congruency effect observed in struggling readers diminishes over time or if it eventually reverts into an incongruency effect as seen in typical readers.

As children train the associations of letters and speech sounds, parts of the word-selective visual cortex rapidly begin to specialize in processing written language (Brem et al., 2010; Dehaene-Lambertz et al., 2018). Parts of vOTC, often referred to as the VWFA, have been shown to preferably respond to words over other categories of visual stimuli (Dehaene et al., 2010). Already after a short artificial grapheme-phoneme training, young prereaders (5–6 years old) show increased neural responses to letter-like symbols in parts of vOTC (Pleisch et al., 2019a). This emerging specialization in vOTC to visually and audiovisually presented written characters has been shown to be performance-dependent, with faster grapheme-phoneme correspondence learning being associated with increased vOTC activation (Karipidis et al., 2017; Pleisch et al., 2019a).

Besides activations in superior temporal areas involved in multisensory processing, our longitudinal analysis confirms the rapid increase in vOTC activation when processing letters after the onset of reading acquisition. Activation in the letter-specific vOTC ROI increased from kindergarten to first grade, with this developmental effect being particularly pronounced in the typical reading group. Text-sensitive parts of the vOTC (VWFA) have been consistently found to respond less to text stimuli in children (van der Mark et al., 2009; Olulade et al., 2015; Brem et al., 2020), adolescents (Kronschnabel et al., 2013), and adults (McCandliss et al., 2003) with dyslexia compared to typical readers. Reduced vOTC activation in children with dyslexia has also been reported during audiovisual processing of syllables (Romanovska et al., 2021). Importantly, visual processing of text in vOTC might also facilitate access to phonological representations through connectivity to other regions, such as the auditory cortex. Disruptions in functional and structural connectivity from vOTC to other regions of the reading network are likely to be associated with impairments in fast word recognition in dyslexia (Richlan, 2019). Here, we provide longitudinal evidence of reading-skill dependent development of vOTC activation during audiovisual processing of single letter-speech sound correspondences. In addition, the observed incongruency effect in the left vOTC suggests that visual areas specialized to process letters and words are sensitive to effects of audiovisual integration during critical periods of learning.

Audiovisual integration effects have been predominantly described in auditory and visual regions, and lesions in the above mentioned temporal and occipital regions have been found to be most disruptive of audiovisual integration processes for speech (Hickok et al., 2018). However, there are frontal and parietal regions involved in reading that may also play a crucial role in letter-speech sound processing (Pugh et al., 2000). We found a congruency effect in the left angular gyrus that was present in first grade and positively associated with later reading skills. Parts of the inferior parietal cortex are involved in cross-modal processing and in semantic processing, including componential analysis of letter-sound associations (Taylor et al., 2014). The engagement of parietal regions may support learning a novel orthography (Quinn et al., 2017) and may reflect less automatized audiovisual processing in beginning readers (Xu et al., 2018). Learning new letter-speech sound correspondences also results in changes of activation patterns in the IFG (Hashimoto and Sakai, 2004). Typical readers showed overall higher activation in the IFG which significantly increased after the start of reading acquisition and showed the largest deviation from the poor reading group at T3. Across all participants and time points, we identified a cluster in the left IFG that responded stronger to incongruent than congruent letter-speech sound pairs, suggesting a strong mismatch response in this region. Supplementary analysis in the left IFG ROI suggested that this incongruency effect increased over time. The IFG has been discussed as an integration site for multisensory information and may be specifically involved in category learning (Li et al., 2020).

Recent fMRI studies have shown a strong convergence of spoken and written language networks in perisylvian and frontal brain regions that appears to be universal for skilled readers of different languages (Rueckl et al., 2015) and already present in beginning readers (Marks et al., 2019). The present study extends this knowledge by providing additional longitudinal evidence for the crucial role of integrating audiovisual information in the early stages of reading acquisition. We found evidence for a growing engagement of auditory, visual, and multisensory integration areas in processing letter-speech sound pairs in the first months of reading acquisition. Although the contribution of familial risk for dyslexia to this development remains unclear, we demonstrate different developmental trajectories between typical and poor readers in the STG, IFG, and vOTC. Future research will clarify how well these developmental effects generalize to less transparent alphabetic languages, such as English. Importantly, we also found a predictive association between early sensitivity to audiovisual congruency in prereading stages and later reading fluency skills. This longitudinal study provides evidence that individual developmental trajectories of letter and speech sound processing are related to children’s reading achievement and advances current knowledge about the development of brain systems for reading.

## Data Availability Statement

The raw data supporting the conclusions of this article will be made available by the authors, upon reasonable request.

## Ethics Statement

The studies involving human participants were reviewed and approved by the Kantonale Ethikkommission Zürich. Written informed consent to participate in this study was provided by the participants’ legal guardian/next of kin.

## Author Contributions

IK, GP, and SB conceptualized study. IK and GP collected the data. IK, GP, SD, and GF-G analyzed the data. SB acquired funding and provided resources. IK made the figures and wrote the manuscript. All authors contributed to the editing of the manuscript and approved the submitted version.

## Conflict of Interest

The authors declare that the research was conducted in the absence of any commercial or financial relationships that could be construed as a potential conflict of interest.

## Publisher’s Note

All claims expressed in this article are solely those of the authors and do not necessarily represent those of their affiliated organizations, or those of the publisher, the editors and the reviewers. Any product that may be evaluated in this article, or claim that may be made by its manufacturer, is not guaranteed or endorsed by the publisher.

## Footnotes

1
www.neurobs.com
