# Supplementary material for: Developmental Trajectories of Letter and Speech Sound Integration During Reading Acquisition
Source: Front Psychol. 2021 Nov 16;12:750491. doi: 10.3389/fpsyg.2021.750491 (PMC8636811; doi:10.3389/fpsyg.2021.750491)
Supplement: Supplementary file 1 [file Table_1.docx]

Supplementary Material

Developmental trajectories of letter and speech sound integration during reading acquisition

Iliana I. Karipidis^1,2^, Georgette Pleisch^1^, Sarah V. Di Pietro^1,3^, Gorka Fraga-González ^1^, Silvia Brem^1,3,4*^

^1^Department of Child and Adolescent Psychiatry and Psychotherapy, University Hospital of Psychiatry Zurich, University of Zurich, Switzerland

^2^Center for Interdisciplinary Brain Sciences Research, Stanford University School of Medicine, Stanford, CA, USA

^3^Neuroscience Center Zurich, University of Zurich and ETH Zurich, Switzerland

^4^MR-Center of the University Hospital of Zurich, University of Zurich, Switzerland

# Supplementary Methods

## Motion correction and scrubbing

For the core sample of n=29, at least one volume was repaired using the ArtRepair toolbox for 12 cases for T1 (m = 6.5 volumes) and T2 (m = 7.2 volumes), and 11 cases for T3 (m = 10.7 volumes). Scrubbing was performed in 10 cases for T1 (m = 6.5 volumes), 8 cases for T2 (m=8 volumes), and 9 cases in T3 (m = 8.3 volumes). Four data sets were not analyzed in full length either because the last blocks exceeded the defined motion thresholds (2 subjects, 5 blocks discarded) or because scanning had to be stopped prematurely (2 subjects, 2 and 3 blocks discarded respectively).

For the enlarged sample of n = 47, at least one volume was repaired for 17/36 cases for T1 (m = 7.5 volumes), 21/45 cases for T2 (m = 7.2 volumes), and 16/40 cases for T3 (m = 9.5 volumes). Scrubbing was performed in 13 cases for T1 (m = 7.4 volumes), T2 (m = 9.2 volumes), and T3 (m = 7.3 volumes).

# Supplementary Analyses

## Letter-speech sound processing in functional ROIs

Additional functional region of interest (ROI) analyses were performed in left vOTC and the left Heschl’s gyrus (HG). For each time point and condition, masks of group activation (against baseline) in the left visual cortex and auditory cortex were extracted (n = 29; voxel-wise FWEcor *P* < .05). ROIs of the left visual and auditory regions were created by collapsing the masks across all time points and all unimodal and bimodal conditions (OR statement). The resulting functional ROIs in the visual cortex were then overlapped with an anatomical mask of the fusiform gyrus (AND statement), and functional ROIs in the auditory cortex were overlapped with an anatomical mask of the Heschl’s gyrus (Suppl. Fig. 2).

The LMM with factors time, congruency, and reading fluency was computed using mean beta values in the left vOTC ROI and in the left HG ROI. The results for the functional left vOTC ROI, confirmed the effects that we found for the letter-specific vOTC ROI. We found a significant main effect of time (F(2,177) = 8.50, P < .0001; Suppl. Fig. 2A) that was characterized by an increase of activation in the left vOTC ROI from T1 to T2 (t(177) = 3.44, Pcor = .002) and a decrease from T2 to T3 (t(177) = 3.53, Pcor = .002). The interaction of time and reading ability was also significant (F(2,177) = 8.76, P = .005; Suppl. Fig. 2A). Activation in the left vOTC during audiovisual processing of letters increased in children with typical reading outcomes from T1 to T2 (t(177) = 4.98, Pcor < .001), and from T1 to T3 (T3>T1 t(177) = 3.58, Pcor = .006). Children with poor reading outcomes did not show a significant difference of activation in the left vOTC from T1 to T2 (t(177) = 0.65, Pcor = .987) but a decrease at T3 (T1>T3: t(177) = 2.58, Pcor = .108; T2>T3: t(177) = 3.49, Pcor = .008). Group differences within time points were not significant (Pcor > .183).

A significant main effect of time was also found for the left HG ROI (F(2,182) = 17.38, P < .001; Suppl. Fig. 2B). Audiovisual processing of letter resulted in stronger activation in the left HG in T2 and T3 compared to T1 (T2>T1: t(182) = 5.89, Pcor < .001; T3>T1: t(182) = 3.60, Pcor = .001). This activation showed a trend towards a decrease from T2 to T3 (t(182) = 2.25, Pcor = .066). We also found a significant interaction of time and reading ability (F(2, 182) = 7.74, P < .001; Suppl. Fig. 2B). Typical readers showed a significant increase of activation in the left HG from T1 to T2 (t(182) = 6.75, Pcor < .001), an increase that persisted at T3 (T3>T1: t(182) = 6.34, Pcor < .001). The group of poor reading children did not show such a clear developmental pattern, with the data suggesting a slight increase of activation from T1 to T2 which was not statistically significant (t(182) = 2.36, Pcor = .177) and showed a trend towards a decrease at T3 (T3>T2: t(182) = 2.74, Pcor = .072). Group comparisons at T1 and T2 were not significant (Pcor> .629) but the diverging development of the two groups at T3 resulted in significantly stronger activation in the left HG for children with typical reading skills than poor reading skills (t(182) = 2.94, Pcor = .043). Our analysis did not reveal a significant main effect of congruency or reading or any further interaction effects in the left vOT or left HG ROI (P > .403).

LMM analyses were repeated using the incongruency effect values for each functional ROI and including the factors time and reading fluency. This analysis revealed no significant effects (Suppl. Fig. 3).

Correlations of ROI-specific incongruency effects within each time point and reading fluency outcomes at T3 were not significant (P > .099). Familial risk for dyslexia was correlated with the incongruency effect in the functional left vOTC ROI at T2, i.e. a higher risk for dyslexia was associated with an increased incongruency effect in vOTC during the first year of formal reading instruction. Letter-sound knowledge at T1 correlated with the incongruency effect in the left HG ROI at T3, i.e. a higher letter-sound knowledge at the prereading stage was associated with stronger incongruency effect in the HG in the second year of formal reading instruction. The correlations were not statistically significant after correction for multiple comparisons.

# Supplementary Figures and Tables

## Supplementary Figures


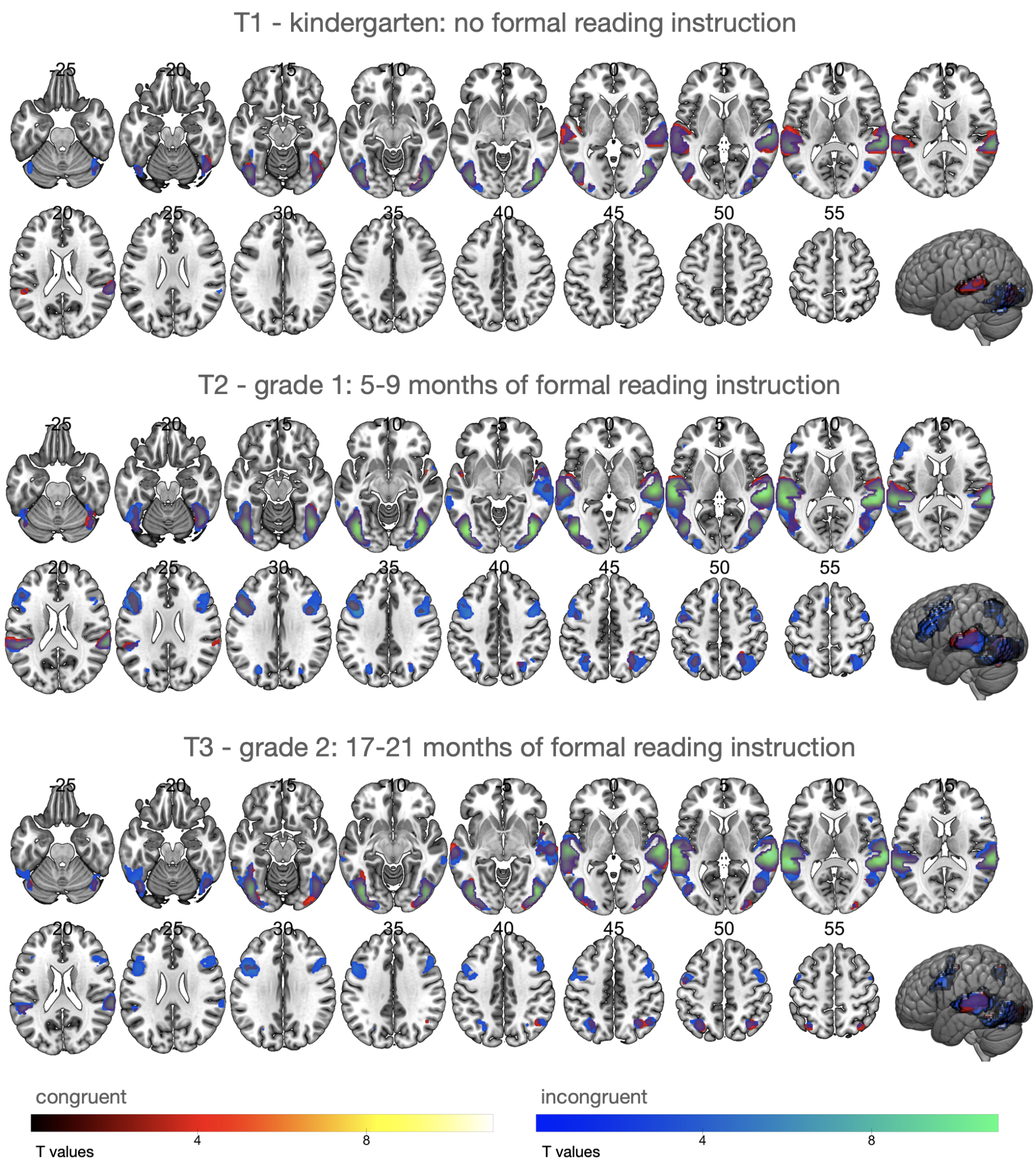


**Supplementary Figure 1.** **Whole-brain fMRI analysis (n=29).** Significant BOLD responses for congruent (warm colors) and incongruent conditions (cold colors) compared to baseline within each time point.

#
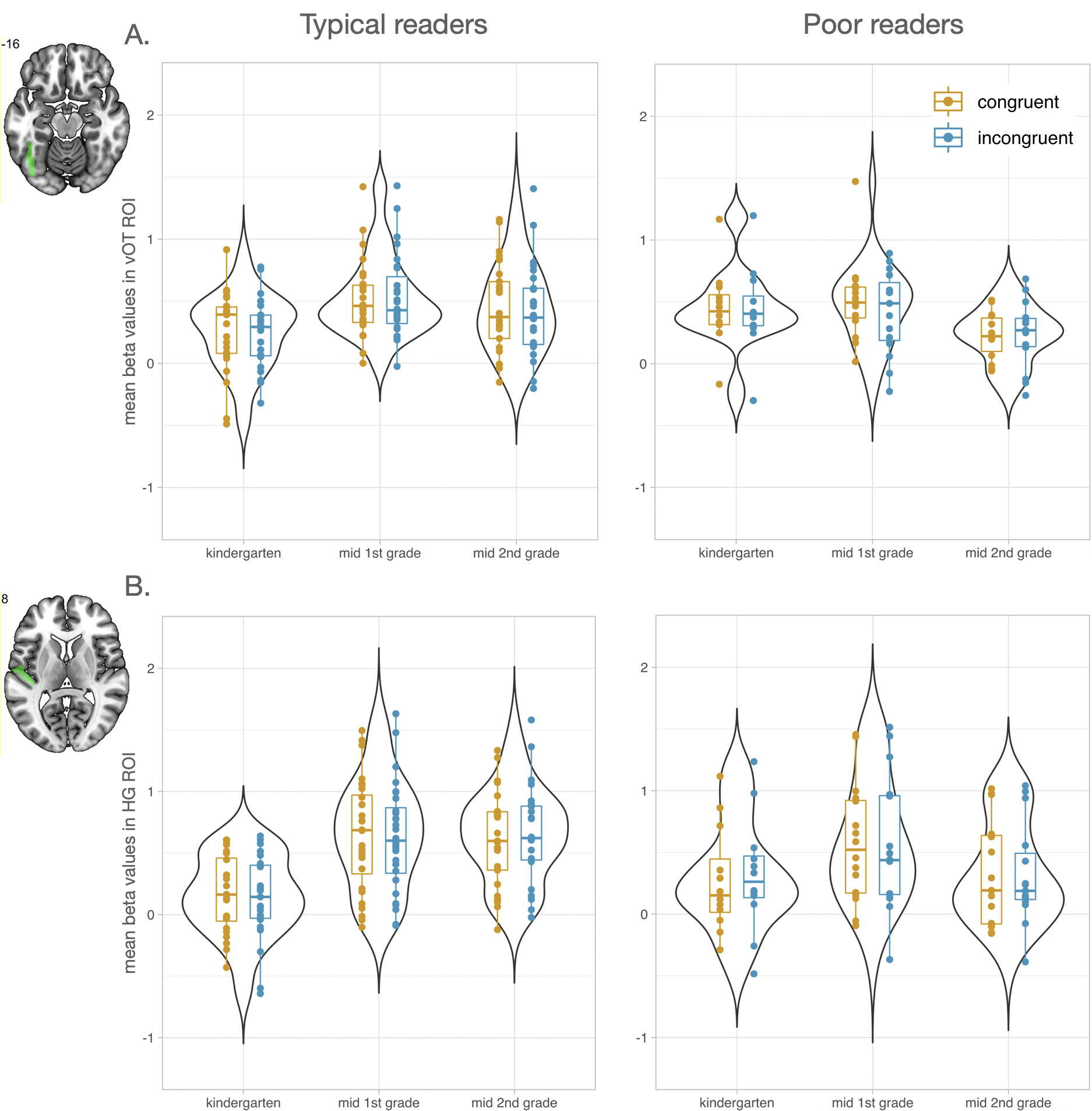


**Supplementary Figure 2. Functional region of interest analyses (n=47).** Mean beta values in ROIs increased from kindergarten (T1) to mid 1st grade (T2) in the typical reading group across both conditions. (A) Mean beta values in vOTC ROI (B) Mean beta values in HG ROI. Left panels show values for typical readers and right panels for poor readers. Mean responses to congruent letter-speech sound pairs are shown in orange and to incongruent in blue.


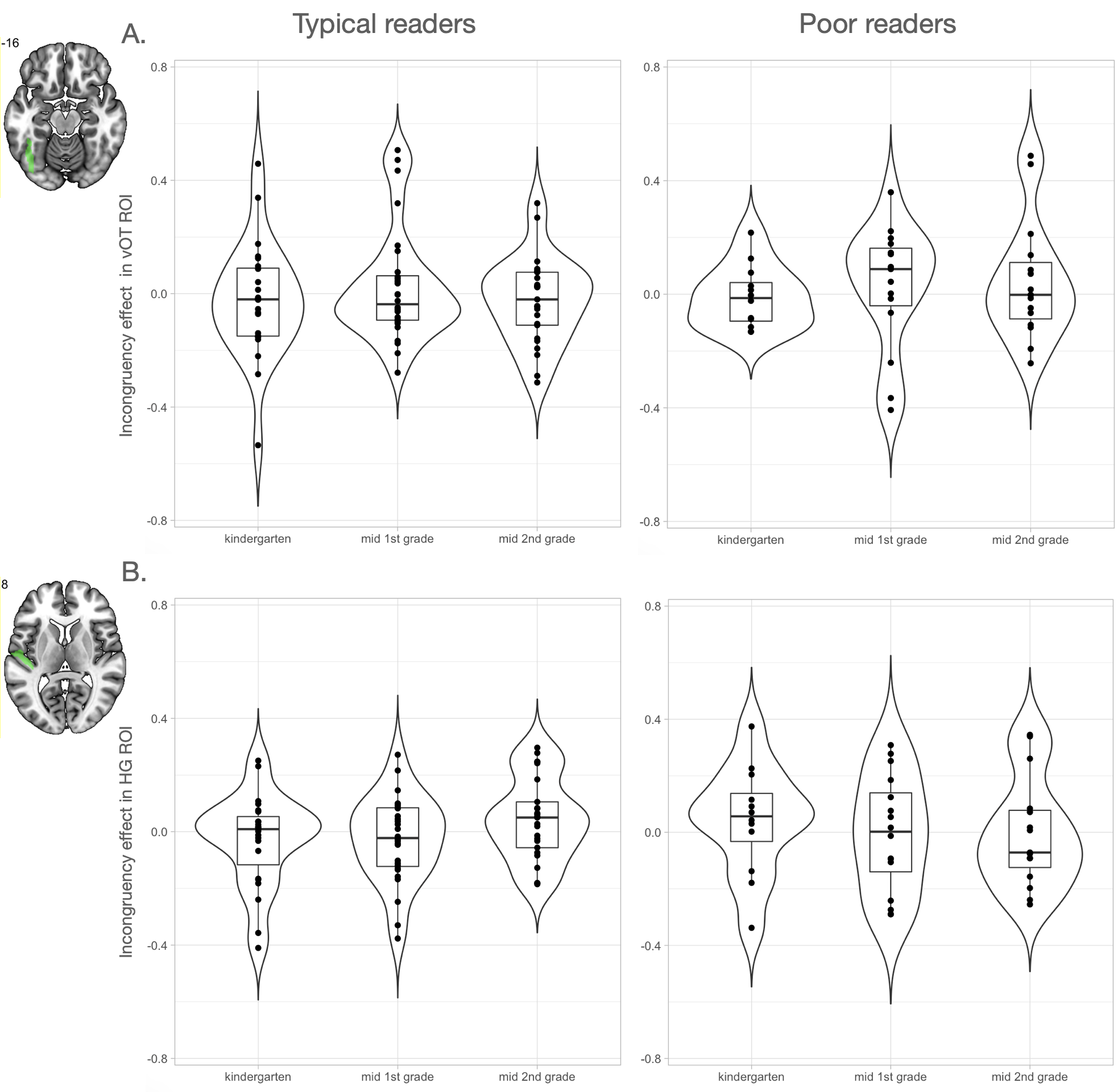


**Supplementary Figure 3.** **Incongruency effect in functional ROIs (n=47).** Positive values represent stronger activation for incongruent than congruent letter-speech sound pairs, and negative values represent stronger activation for congruent than incongruent pairs. The LMM with the difference values (incongruent-congruent) revealed no significant effect of time or reading ability (P > .286) in the left vOT ROI (A) and left HG ROI (B).


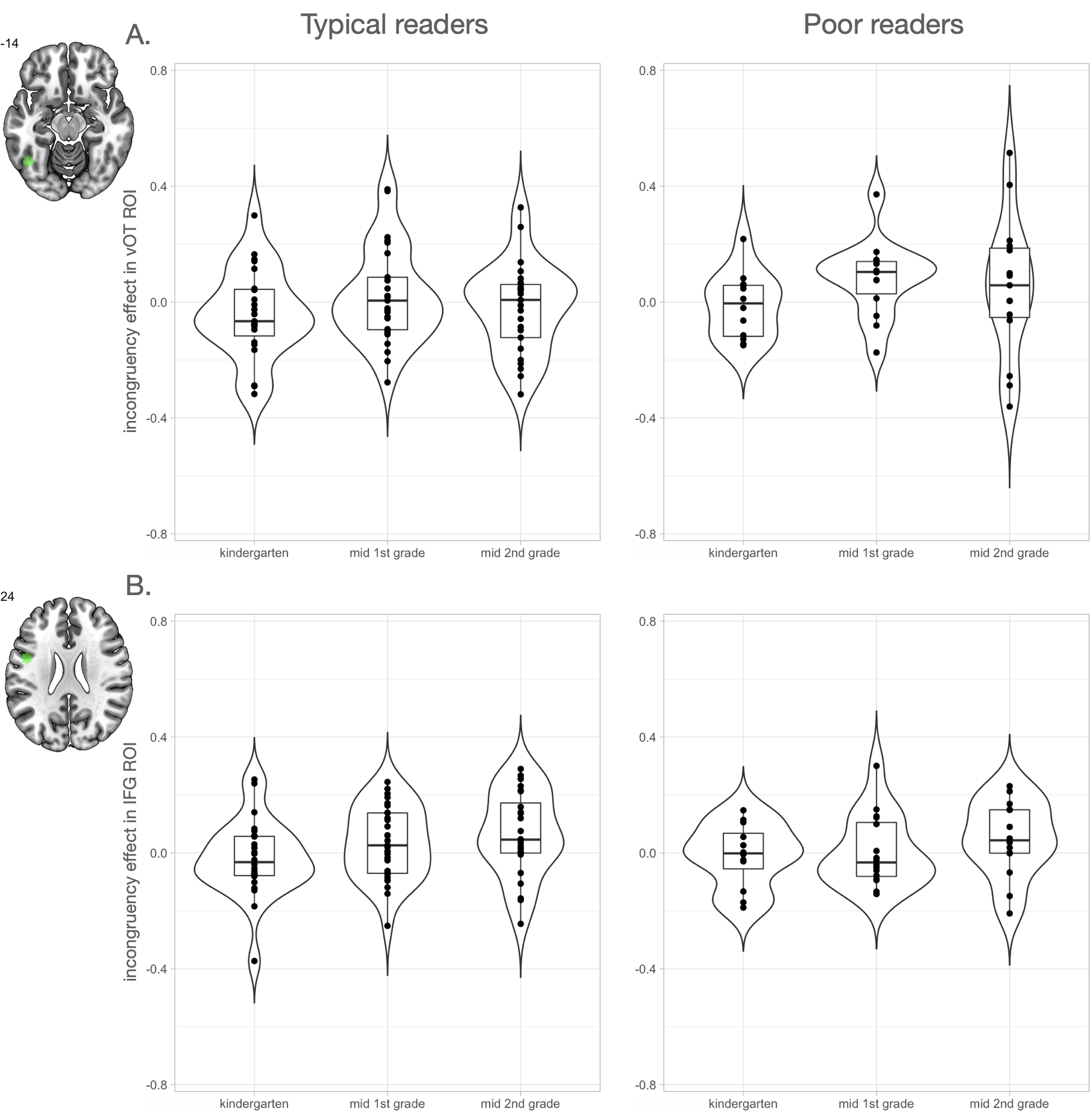


**Supplementary Figure 4.** **Incongruency effect in letter-specific ROIs (n=47).** Positive values represent stronger activation for incongruent than congruent letter-speech sound pairs, and negative values represent stronger activation for congruent than incongruent pairs. The LMM with the difference values (incongruent-congruent) revealed (A) a significant effect of time in the left vOTC ROI (F(67,2) = 3.25, P = .045, T2 > T1 t(67) = 2.54, Pcor = .036) and (B) a trend in the left IFG ROI (B; F(67,2) = 2.84, P = .065, T3 > T1 t(67) = 2.38, Pcor = .052). For both ROIs, the main effect or reading ability (P > .150) and the interaction of reading ability and time were not significant (P > .739).


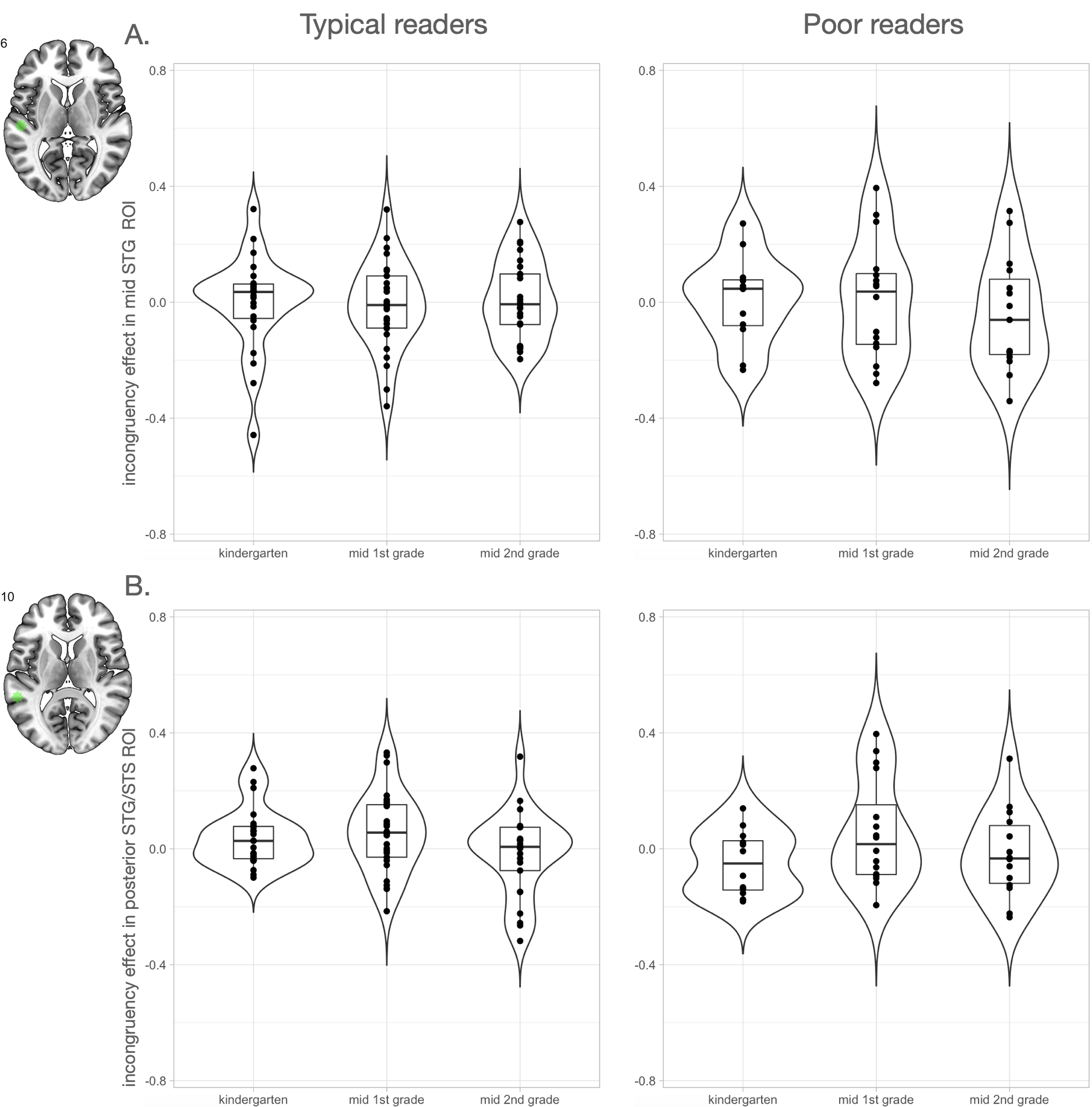


**Supplementary Figure 5.** **Incongruency effect in audiovisual ROIs (n=47).** Positive values represent stronger activation for incongruent than congruent letter-speech sound pairs, and negative values represent stronger activation for congruent than incongruent pairs. The LMM with the difference values (incongruent-congruent) revealed a significant effect of time in (B) the left posterior ROI (F(68,2) = 3.17, P = .048) and not in (A) the left mid STG ROI (A; F(68,2) = 0.26, P = .774). Activation in the left posterior STG remained similar from T1 to T2 (t(68) = 1.89, Pcor = .148) but tended to decrease from T2 to T3 (t(68) = 2.33, Pcor = .058). For both ROIs, the main effect or reading ability (P > .301) and the interaction of reading ability and time were not significant (P > .354).

## Supplementary Table

| *brain area* | *MNI coordinates* | | | *Voxels* | *T-value* | *Peak-level*  *P uncor* | *cluster-level P uncor* | *cluster-level P FWEcorr* |
| --- | --- | --- | --- | --- | --- | --- | --- | --- |
|  | **x** | **y** | **z** |  |  |  |  |  |
| **T1 congruent** | | | | | | | | |
| vOTC right | 43 | -78 | -6 | 623 | 8.61 | <0.001 | <0.001 | <0.001 |
| STG right | 61 | -33 | 12 | 433 | 6.98 | <0.001 | <0.001 | <0.001 |
| vOTC left | -41 | -72 | -12 | 474 | 6.80 | <0.001 | <0.001 | <0.001 |
| STG left | -59 | -39 | 12 | 333 | 6.36 | <0.001 | <0.001 | <0.001 |
| **T1 incongruent** | | | | | | | | |
| vOTC right | 40 | -78 | -6 | 571 | 8.41 | <0.001 | <0.001 | <0.001 |
| STG right | 64 | -30 | 9 | 498 | 8.02 | <0.001 | <0.001 | <0.001 |
| vOTC left | -44 | -75 | -12 | 384 | 6.92 | <0.001 | <0.001 | <0.001 |
| STG left | -59 | -39 | 12 | 469 | 6.66 | <0.001 | <0.001 | <0.001 |
| **T2 congruent** | | | | | | | | |
| vOTC/STG right | 64 | -30 | 12 | 1461 | 10.38 | <0.001 | <0.001 | <0.001 |
| vOTC/STG left | -59 | -27 | 6 | 1418 | 9.38 | <0.001 | <0.001 | <0.001 |
| MFG/IFG left | -47 | 15 | 33 | 187 | 5.00 | <0.001 | <0.001 | 0.001 |
| PrG left | -44 | -3 | 48 | 35 | 4.62 | <0.001 | 0.048 | 0.360 |
| SPL/AnG right | 28 | -51 | 48 | 83 | 4.56 | <0.001 | 0.005 | 0.041 |
| SPL/AnG left | -32 | -63 | 51 | 51 | 4.40 | <0.001 | 0.020 | 0.172 |
| MFG right | 43 | 6 | 30 | 38 | 4.08 | <0.001 | 0.041 | 0.314 |
| PrG right | 49 | -3 | 51 | 18 | 3.94 | <0.001 | 0.143 | 0.733 |
| **T2 incongruent** | | | | | | | | |
| vOTC/STG right | 58 | -24 | 3 | 1647 | 10.72 | <0.001 | <0.001 | <0.001 |
| vOTC/STG left | -41 | -78 | -6 | 1697 | 10.09 | <0.001 | <0.001 | <0.001 |
| MFG/IFG left | -50 | 15 | 30 | 601 | 7.06 | <0.001 | <0.001 | <0.001 |
| MFG/IFG right | 43 | 6 | 33 | 368 | 5.91 | <0.001 | <0.001 | <0.001 |
| SPL/AnG left | -35 | -63 | 54 | 250 | 5.61 | <0.001 | <0.001 | <0.001 |
| SPL/AnG right | 31 | -57 | 45 | 274 | 5.31 | <0.001 | <0.001 | <0.001 |
| SMC left | -5 | 21 | 51 | 42 | 4.23 | <0.001 | 0.033 | 0.261 |
| **T3 congruent** | | | | | | | | |
| STG left | -56 | -39 | 12 | 760 | 10.32 | <0.001 | <0.001 | <0.001 |
| STG right | 61 | -27 | 6 | 716 | 10.30 | <0.001 | <0.001 | <0.001 |
| vOTC right | 43 | -75 | -6 | 513 | 8.78 | <0.001 | <0.001 | <0.001 |
| vOTC left | -41 | -72 | -9 | 502 | 7.54 | <0.001 | <0.001 | <0.001 |
| AnG right | 34 | -66 | 51 | 111 | 4.75 | <0.001 | 0.001 | 0.013 |
| AnG/SPL left | -32 | -66 | 51 | 65 | 4.34 | <0.001 | 0.010 | 0.091 |
| PrG left | -53 | -3 | 51 | 17 | 4.16 | <0.001 | 0.154 | 0.758 |
| MFG left | -44 | 12 | 30 | 19 | 3.52 | <0.001 | 0.133 | 0.708 |
| **T3 incongruent** | | | | | | | | |
| STG/vOTC right | 64 | -30 | 9 | 1488 | 10.22 | <0.001 | <0.001 | <0.001 |
| STG/vOTC left | -59 | -36 | 9 | 1569 | 9.70 | <0.001 | <0.001 | <0.001 |
| PrG/IFG/MFG left | -53 | 0 | 48 | 320 | 5.35 | <0.001 | <0.001 | <0.001 |
| AnG/SPL left | -32 | -63 | 51 | 115 | 4.78 | <0.001 | 0.001 | 0.011 |
| PrG/IFG/MFG right | 49 | 21 | 36 | 253 | 4.36 | <0.001 | <0.001 | <0.001 |
| AnG/SPL right | 31 | -60 | 45 | 105 | 4.33 | <0.001 | 0.002 | 0.017 |
| SMC left | -5 | 0 | 63 | 21 | 3.85 | <0.001 | 0.116 | 0.657 |

**Supplementary Table 1.** vOTC = ventral occipitotemporal cortex; STG = superior temporal gyrus; MFG = middle frontal gyrus; IFG = inferior frontal gyrus; PrG = precentral gyrus; SPL = superior parietal lobule; AnG = angular gyrus; SMC = supplementary motor cortex
